# Genome-Wide Analysis of the *G2-Like* Transcription Factor Genes and Their Expression in Different Senescence Stages of Tobacco (*Nicotiana tabacum* L.)

**DOI:** 10.3389/fgene.2021.626352

**Published:** 2021-05-31

**Authors:** Mingyue Qin, Binghui Zhang, Gang Gu, Jiazheng Yuan, Xuanshong Yang, Jiahan Yang, Xiaofang Xie

**Affiliations:** ^1^College of Life Sciences, Fujian Agriculture and Forestry University, Fuzhou, China; ^2^Institute of Tobacco Science, Fujian Provincial Tobacco Company, Fuzhou, China; ^3^Department of Biological and Forensic Sciences, Fayetteville State University, Fayetteville, NC, United States; ^4^Fujian Key Laboratory of Crop Breeding by Design, Fujian Agriculture and Forestry University, Fuzhou, China

**Keywords:** gene expression, senescence, phylogenetic analysis, *G2-like* transcription factors, *Nicotiana tobacum*

## Abstract

The *Golden2-like* (GLK) transcription factors play important roles in regulating chloroplast growth, development, and senescence in plants. In this study, a total of 89 *NtGLK* genes (*NtGLK1–NtGLK89*) were identified in the tobacco genome and were classified into 10 subfamilies with variable numbers of exons and similar structural organizations based on the gene structure and protein motif analyses. Twelve segmental duplication pairs of *NtGLK* genes were identified in the genome. These *NtGLK* genes contain two conserved helix regions related to the HLH structure, and the sequences of the first helix region are less conserved than that of the second helix motif. *Cis*-regulatory elements of the *NtGLK* promoters were widely involved in light responsiveness, hormone treatment, and physiological stress. Moreover, a total of 206 *GLK* genes from tomato, tobacco, maize, rice, and *Arabidopsis* were retrieved and clustered into eight subgroups. Our gene expression analysis indicated that *NtGLK* genes showed differential expression patterns in tobacco leaves at five senescence stages. The expression levels of six *NtGLK* genes in group C were reduced, coinciding precisely with the increment of the degree of senescence, which might be associated with the function of leaf senescence of tobacco. Our results have revealed valuable information for further functional characterization of the *GLK* gene family in tobacco.

## Introduction

The GOLDEN2-LIKE (GLK) proteins are included in the GARP (Golden2, ARR-B and Psr1) domain superfamily of transcription factors (TFs) (Riechmann et al., 2000; Xiao et al., 2019). The GLK transcription factor was originally identified in the C4 plant of maize (*Zea mays* L.) (Hall et al., 1998). A typical GLK protein usually contains two conserved domains, namely, a Myb-DNA binding domain (DBD) and a C-terminal (GCT) box (Rossini et al., 2001).

Members of the GLK family play important roles in the formation and development of chloroplasts (Rossini et al., 2001; Fitter et al., 2002; Waters et al., 2008; Powell et al., 2012; Jarvis and López-Juez, 2013) and have been involved in the various defense processes of organisms, including biotic and abiotic stresses (Savitch et al., 2007; Schreiber et al., 2011; Murmu et al., 2014; Han et al., 2016; Nagatoshi et al., 2016). In *Arabidopsis* (*Arabidopsis thaliana*), the *AtGLK1* and *AtGLK2* genes act redundantly to regulate chloroplast development (Fitter et al., 2002; Yasumura et al., 2005). *AtGLK1* overexpression enhances resistance to the pathogen *Fusarium graminearum* (Savitch et al., 2007; Schreiber et al., 2011) and increases susceptibility to the virulent oomycete pathogen *Hyaloperonospora arabidopsidis* (*Hpa*) (Murmu et al., 2014), whereas the *glk1 glk2* double mutant increases the resistance to the *Hpa* gene compared to that of the wild type (Murmu et al., 2014). In addition, it has been reported that GLKs can interact with ANAC092 (ORE-1) to regulate leaf senescence (Rauf et al., 2013). In tomato (*Solanum lycopersicum*), the overexpression of *GLK* increases the expressions of genes related to chloroplast development and fruit photosynthesis, and these changes result in the enhancement of the carbohydrate and carotenoid levels in ripe fruits (Powell et al., 2012). In maize, *ZmGlk1* has been considered to play important roles in the chloroplast development of mesophyll cell in C4 plant tissues (Rossini et al., 2001; Fitter et al., 2002).

The senescence of tobacco (*Nicotiana tabacum*) leaves is a positive and orderly process involving the transformation and mobilization of nutrients. As a model plant, the study of the plant senescence and internal material transport rules in tobacco has special significance in the research of plant physiology and development (Gregersen et al., 2013). During the phase of leaf senescence, leaf cells undergo orderly changes in structure, metabolism, and gene expression, along with a series of degradations, including the chlorophyll content depletion and diminishing photosynthetic capacity (Lira et al., 2017; Schippers et al., 2015; Gan and Amasino, 1997; Lim et al., 2003). Normally, leaf color change is the most intuitive phenomenon, which is due to the degradation of chlorophyll in chloroplasts (Hörtensteiner, 2006; Kräutler, 2016); therefore, the degree of etiolation has usually been used as an important criterion to assess the senescence of leaves. Because of the importance of the members of the *GLK* gene family for the development of chloroplasts (Fitter et al., 2002; Waters et al., 2008; Powell et al., 2012) and its association with leaf senescence (Rauf et al., 2013), it is important to investigate this gene family and assess the relationship between gene expression and senescence in leaf.

The *GLK* gene family has been identified and characterized in several plant species, including maize (*Zea mays* L.) (Liu et al., 2016) and tomato (Liu, 2018). However, the *GLK* gene family has not been thoroughly examined in tobacco, to the best of our knowledge. A comprehensive investigation of all the *GLK* genes with current tobacco genome sequence data, including the family members and the detailed organization of the gene sequences in tobacco, should be conducted. The objectives of this study were to analyze the *GLK* gene family including the *GLK* gene structure, chromosomal localization, and phylogenetic relationship in the tobacco genome and to reveal the expression regulation of the *GLK* gene family members at different senescence stages of tobacco leaves. The information derived from this study will be useful for further functional exploration on the *GLK* gene family in tobacco.

## Materials and Methods

### Identification of *GLK* Genes in Tobacco

The published GLK protein sequences of maize (Liu et al., 2016), rice (*Oryza sativa*) (Rossini et al., 2001), and *A. thaliana* (Fitter et al., 2002) were used as query sequences to identify the GLK proteins in tobacco using the BLASTP tool and the tobacco genome sequences (Edwards et al., 2017) in the Sol Genomics Network database^1^. More than 30% similarity and an *E* value less than *E*^––10^ were set as the parameters to define the tobacco candidate GLK proteins. The domains of all the candidate GLK proteins of tobacco were checked using the Conserved Domain Database (CDD) tool^2^ (Lu et al., 2020). Finally, the sequences with complete GLK domains were retained and were renamed (*NtGLK*). Detailed information of the *NtGLK* genes, including the gene IDs, physical position, sequences of the genes and proteins, and the coding sequences (CDS), were retrieved from the Sol Genomics Network database^3^. The features of the NtGLK proteins were calculated using online ExPASy programs^4^ (Bjellqvist et al., 1993; Bjellqvist et al., 1994; Wilkins et al., 1999).

### Multiple Sequence Alignment and Phylogenetic Analysis

The amino acid sequences of all the tobacco GLK proteins were aligned using the ClustalX1.83 tool (Thompson et al., 1997). The alignment of the NtGLK protein conserved domain sequences was exhibited by DNAMAN^5^ (Altschul et al., 1990). A phylogenetic tree with 1,000 bootstrap replicates was generated using the neighbor-joining method of the MEGAX software (Kumar et al., 2018). The classifications of tobacco GLK proteins were determined according to the topology and bootstrap values of the phylogenetic tree.

### Chromosomal Location and Gene Duplication

Information on the physical position image of the *NtGLK* genes was obtained based on the MapInspect tool^6^. To investigate gene duplication, the criteria for the proportion of overlap and the similarity between the two sequences were set to be >70% (Gu et al., 2002; Yang et al., 2008). Segmental duplication and tandem duplication were defined based on the method reported by Wang et al. (2010).

### Gene Structure and Conserved Motif Identification

The gene structures of the *NtGLK* genes were identified by the GSDS^7^ platform using the complete sequence of the genomic sequence and CDS for *NtGLK* downloaded from the tobacco genome (Hu et al., 2015). The conserved motifs of the NtGLK proteins were analyzed by the MEME program^8^ (Ma et al., 2014), and the optimum motif width and the maximum number of motifs were set to 5–100 and 20 residues, respectively, with the remaining parameters in default. Motif annotation was identified using the CDD tools. About 1,500 bp of DNA sequence upstream of the starting codon of the *NtGLK* genes were extracted from the tobacco genome to decipher the *cis*-elements with the online tool PlantCARE^9^ (Lescot et al., 2002) for *cis*-element prediction and the TBtools software (Chen et al., 2020) for the visualization.

### *NtGLK* Expression Analysis in Different Senescence Stages of Tobacco Leaves

*Nicotiana tabacum* cv. Cuibi 1 (CB-1) was used in this study. According to the morphological characteristics of leaf color, vein, and villus (Xu et al., 2017), five senescence stages (M1, M2, M3, M4, and M5) of the middle leaves (eighth to 10th, counted from the bottom to the top) were collected in the same field as samples. Three biological replicates with each replicate containing three leaves from different plant were collected. Total RNA was extracted using a total RNA isolation kit (PR2401, Bioteke Corporation, China). A total of 15 RNA samples were sequenced on Illumina HiSeq 2000 performed by BioMarker Technologies^10^ (BioMarker, Beijing, China). The gene expression level was assessed according to the FPKM (fragments per kilobase of transcript sequence per million base pairs sequenced) value (Trapnell et al., 2010). The complementary DNA (cDNA) samples were synthesized by a SMART cDNA synthesis kit, and quantitative reverse transcriptase (qRT-PCR) reactions were conducted with the SYBR Premix Ex Taq based on the manufacturer’s instruction (Takara). The primers used for qRT-PCR analysis are listed in Supplementary Table 1. The specific exon regions of the target genes were used for primer design. The primer pair of each target gene was also prescreened to ensure the uniqueness of the amplification product. Three biological replicates and three technical replicates were performed. The expression level of each selected *NtGLK* gene in the M1 stage was used as the control, while the relative expression level of the *NtGLK* genes in the different senescence stages was calculated using the 2^−ΔΔCt^ method (Livak and Schmittgen, 2001). A *t* test was conducted to assess the expression differences from the M1 stage to the M2, M3, M4, and M5 stages. Significant difference was set as *p* < 0.05 or *p* < 0.01.

## Results

### Characterization of *GLK* Genes in Tobacco

A BLASTP search was performed using 117 known GLK protein sequences as the query sequences against the tobacco genome database to analyze the *GLK* genes in tobacco, including 59 from maize (Liu et al., 2016), 54 from tomato (Liu et al., 2016), two from rice (Rossini et al., 2001), and two from *A. thaliana* (Fitter et al., 2002). A total of 89 *GLK* genes were identified in tobacco, and these genes were named *NtGLK1* through *NtGLK89*. Detailed information of these genes and their corresponding proteins are listed in Table 1, including the gene ID, gene location, number of exons, protein length (amino acids, aa), molecular mass (MS), and p*I*. The *NtGLK* genes showed a wide range of amino acid sequence lengths and molecular weights. The amino acid residues of NtGLK proteins were oscillating from 114 aa (*NtGLK31*) to 779 aa (*NtGLK41*), with an average of 408 amino acids, whereas the molecular mass was from 13,358.46 Da (*NtGLK31*) to 85,665.25 Da (*NtGLK41*) and the theoretical isoelectric points (p*I*) range from 4.81 (*NtGLK49*) to 9.91 (*NtGLK31*).

**TABLE 1:** The *GLK* gene family in *Nicotiana tabacum* L.

Gene name	Gene ID	Chr./scaffold	Exon	Protein length (aa)	Molecular weight (Da)	p*I*
*NtGLK1*	Nitab4.5_0000016g0360.1	Nt04	12	588	67,407.59	6.82
*NtGLK2*	Nitab4.5_0000058g0230.1	Nt04	6	391	44,736.55	8.72
*NtGLK3*	Nitab4.5_0000061g0100.1	Nitab4.5_0000061	7	464	51,107.17	5.92
*NtGLK4*	Nitab4.5_0000102g0300.1	Nt17	2	334	36,628.15	5.95
*NtGLK5*	Nitab4.5_0000109g0060.1	Nt04	6	415	47,021.29	9.26
*NtGLK6*	Nitab4.5_0000109g0160.1	Nt04	6	281	31,575.53	8.56
*NtGLK7*	Nitab4.5_0000123g0580.1	Nt24	1	300	32,867.68	6.45
*NtGLK8*	Nitab4.5_0000147g0130.1	Nitab4.5_0000147	6	316	34,387.78	5.95
*NtGLK9*	Nitab4.5_0000388g0040.1	Nt24	7	362	40,301.64	8.58
*NtGLK10*	Nitab4.5_0000440g0010.1	Nt03	5	376	42,086.47	6.78
*NtGLK11*	Nitab4.5_0000444g0200.1	Nt24	5	312	35,385.97	8.99
*NtGLK12*	Nitab4.5_0000463g0190.1	Nt22	1	311	34,694.66	5.95
*NtGLK13*	Nitab4.5_0000476g0270.1	Nitab4.5_0000476	7	473	52,022.22	5.8
*NtGLK14*	Nitab4.5_0000543g0010.1	Nt23	6	255	28,440.86	6.67
*NtGLK15*	Nitab4.5_0000570g0280.1	Nitab4.5_0000570	5	510	57,147.73	6.45
*NtGLK16*	Nitab4.5_0000605g0080.1	Nt17	6	419	48,594.05	8.53
*NtGLK17*	Nitab4.5_0000621g0130.1	Nt02	7	484	52,492.13	6.11
*NtGLK18*	Nitab4.5_0000629g0030.1	Nt07	6	655	72,665.22	5.8
*NtGLK19*	Nitab4.5_0000672g0080.1	Nitab4.5_0000672	5	380	41,716.93	6.61
*NtGLK20*	Nitab4.5_0000676g0220.1	Nt15	6	297	32,499.06	7.58
*NtGLK21*	Nitab4.5_0000736g0030.1	Nitab4.5_0000736	5	415	47,115.15	6.25
*NtGLK22*	Nitab4.5_0000850g0070.1	Nt20	6	247	26,811.33	5.61
*NtGLK23*	Nitab4.5_0000916g0020.1	Nt12	4	582	63,275.73	5.99
*NtGLK24*	Nitab4.5_0000969g0020.1	Nt06	6	390	44,622.39	8.44
*NtGLK25*	Nitab4.5_0001083g0040.1	Nt04	3	558	61,358.02	8.95
*NtGLK26*	Nitab4.5_0001088g0210.1	Nt11	6	427	47,155.25	7.79
*NtGLK27*	Nitab4.5_0001094g0080.1	Nt09	5	667	73,495.12	7.27
*NtGLK28*	Nitab4.5_0001097g0090.1	Nt13	6	678	74,931.11	5.97
*NtGLK29*	Nitab4.5_0001164g0090.1	Nitab4.5_0001164	4	420	46,197.33	6.6
*NtGLK30*	Nitab4.5_0001318g0010.1	Nt05	5	545	60,889.33	6.17
*NtGLK31*	Nitab4.5_0001409g0040.1	Nitab4.5_0001409	4	114	13,358.46	9.91
*NtGLK32*	Nitab4.5_0001416g0010.1	Nt07	6	291	32,167.27	6.01
*NtGLK33*	Nitab4.5_0001534g0020.1	Nitab4.5_0001534	7	297	32,419.29	6.46
*NtGLK34*	Nitab4.5_0001632g0010.1	Nt08	6	405	45,548.54	9.02
*NtGLK35*	Nitab4.5_0001663g0280.1	Nt23	5	276	30,025.02	6.86
*NtGLK36*	Nitab4.5_0001777g0040.1	Nt16	6	323	37,180.25	7.66
*NtGLK37*	Nitab4.5_0001933g0010.1	Nitab4.5_0001933	3	338	37,900.5	6.14
*NtGLK38*	Nitab4.5_0002024g0040.1	Nt02	6	296	33,617.42	9.18
*NtGLK39*	Nitab4.5_0002055g0150.1	Nt23	11	563	62,891.85	6.06
*NtGLK40*	Nitab4.5_0002076g0010.1	Nt14	5	278	32,753.05	9.76
*NtGLK41*	Nitab4.5_0002117g0090.1	Nitab4.5_0002117	7	779	85,665.25	6.48
*NtGLK42*	Nitab4.5_0002389g0010.1	Nt22	7	464	51,049.8	5.01
*NtGLK43*	Nitab4.5_0002462g0050.1	Nitab4.5_0002462	5	398	44,288.65	9.23
*NtGLK44*	Nitab4.5_0002465g0020.1	Nt13	6	430	47,599.6	7.09
*NtGLK45*	Nitab4.5_0002606g0020.1	Nt05	1	304	33,054.03	6.54
*NtGLK46*	Nitab4.5_0002803g0020.1	Nitab4.5_0002803	5	654	71,278.23	6.3
*NtGLK47*	Nitab4.5_0002948g0070.1	Nitab4.5_0002948	8	296	32,331.24	6.26
*NtGLK48*	Nitab4.5_0003100g0090.1	Nitab4.5_0003100	11	570	63,380.74	6.24
*NtGLK49*	Nitab4.5_0003295g0220.1	Nt22	4	303	32,814.65	4.81
*NtGLK50*	Nitab4.5_0003484g0070.1	Nitab4.5_0003484	5	493	55,301.42	6.26
*NtGLK51*	Nitab4.5_0003610g0020.1	Nitab4.5_0003610	7	440	49,557.81	6.47
*NtGLK52*	Nitab4.5_0003711g0030.1	Nt21	5	283	33,229.33	9.11
*NtGLK53*	Nitab4.5_0003726g0010.1	Nitab4.5_0003726	5	398	44,328.48	8.75
*NtGLK54*	Nitab4.5_0003836g0060.1	Nitab4.5_0003836	5	301	33,712.65	9.77
*NtGLK55*	Nitab4.5_0003856g0030.1	Nt18	5	327	37,450.18	8.2
*NtGLK56*	Nitab4.5_0003889g0040.1	Nitab4.5_0003889	6	663	73,098.64	7.25
*NtGLK57*	Nitab4.5_0004143g0010.1	Nitab4.5_0004143	6	297	33,751.65	9.11
*NtGLK58*	Nitab4.5_0004327g0030.1	Nitab4.5_0004327	11	563	62,486.11	6.3
*NtGLK59*	Nitab4.5_0004550g0020.1	Nt19	6	601	68,120.64	5.4
*NtGLK60*	Nitab4.5_0004560g0010.1	Nitab4.5_0004560	6	563	62,408.53	5.33
*NtGLK61*	Nitab4.5_0004658g0020.1	Nitab4.5_0004658	11	568	63,109.41	6.38
*NtGLK62*	Nitab4.5_0004835g0040.1	Nt08	5	261	29,209.91	9.43
*NtGLK63*	Nitab4.5_0004892g0040.1	Nitab4.5_0004892	2	335	36,619.21	6.26
*NtGLK64*	Nitab4.5_0004991g0030.1	Nt05	8	478	54,910.12	5.77
*NtGLK65*	Nitab4.5_0005180g0030.1	Nitab4.5_0005180	6	670	73,859.9	5.97
*NtGLK66*	Nitab4.5_0005194g0010.1	Nitab4.5_0005194	5	398	43,657.14	6.98
*NtGLK67*	Nitab4.5_0005233g0020.1	Nitab4.5_0005233	6	573	63,383.99	5.68
*NtGLK68*	Nitab4.5_0006031g0030.1	Nitab4.5_0006031	5	195	21,865.34	6.5
*NtGLK69*	Nitab4.5_0006133g0010.1	Nitab4.5_0006133	7	334	36,245.31	6.27
*NtGLK70*	Nitab4.5_0006324g0050.1	Nitab4.5_0006324	6	339	46,485.81	9.13
*NtGLK71*	Nitab4.5_0006629g0020.1	Nitab4.5_0006629	7	443	48,598	5.68
*NtGLK72*	Nitab4.5_0006900g0010.1	Nitab4.5_0006900	6	294	33,016.07	9.11
*NtGLK73*	Nitab4.5_0006916g0030.1	Nitab4.5_0006916	1	311	34,696.8	6.42
*NtGLK74*	Nitab4.5_0006963g0020.1	Nt20	3	244	26,200.36	6.59
*NtGLK75*	Nitab4.5_0006980g0060.1	Nitab4.5_0006980	7	455	49,707.14	5.06
*NtGLK76*	Nitab4.5_0007123g0020.1	Nitab4.5_0007123	7	440	50,669.27	5.73
*NtGLK77*	Nitab4.5_0007572g0030.1	Nitab4.5_0007572	6	332	37,365.71	9.66
*NtGLK78*	Nitab4.5_0007848g0010.1	Nitab4.5_0007848	7	351	39,045.15	7.74
*NtGLK79*	Nitab4.5_0008054g0010.1	Nitab4.5_0008054	7	333	36,183.32	6.22
*NtGLK80*	Nitab4.5_0008332g0020.1	Nitab4.5_0008332	1	300	32,941.71	6.45
*NtGLK81*	Nitab4.5_0008336g0010.1	Nitab4.5_0008336	4	421	46,640.87	6.54
*NtGLK82*	Nitab4.5_0008908g0020.1	Nitab4.5_0008908	7	273	31,074.39	9.15
*NtGLK83*	Nitab4.5_0009217g0030.1	Nitab4.5_0009217	5	294	33,105.58	9.47
*NtGLK84*	Nitab4.5_0010430g0010.1	Nitab4.5_0010430	6	601	67,954.72	5.69
*NtGLK85*	Nitab4.5_0010689g0010.1	Nitab4.5_0010689	6	488	53,357.22	6.29
*NtGLK86*	Nitab4.5_0011083g0010.1	Nitab4.5_0011083	7	407	46,360.82	7.64
*NtGLK87*	Nitab4.5_0012578g0010.1	Nitab4.5_0012578	5	342	38,135.65	6.07
*NtGLK88*	Nitab4.5_0012878g0020.1	Nitab4.5_0012878	5	186	21,113.57	6.31
*NtGLK89*	Nitab4.5_0014621g0010.1	Nitab4.5_0014621	7	466	51,325.56	6.26

### Structure and Phylogenetic Tree of Tobacco *GLK* Gene Members

**FIGURE 1:**
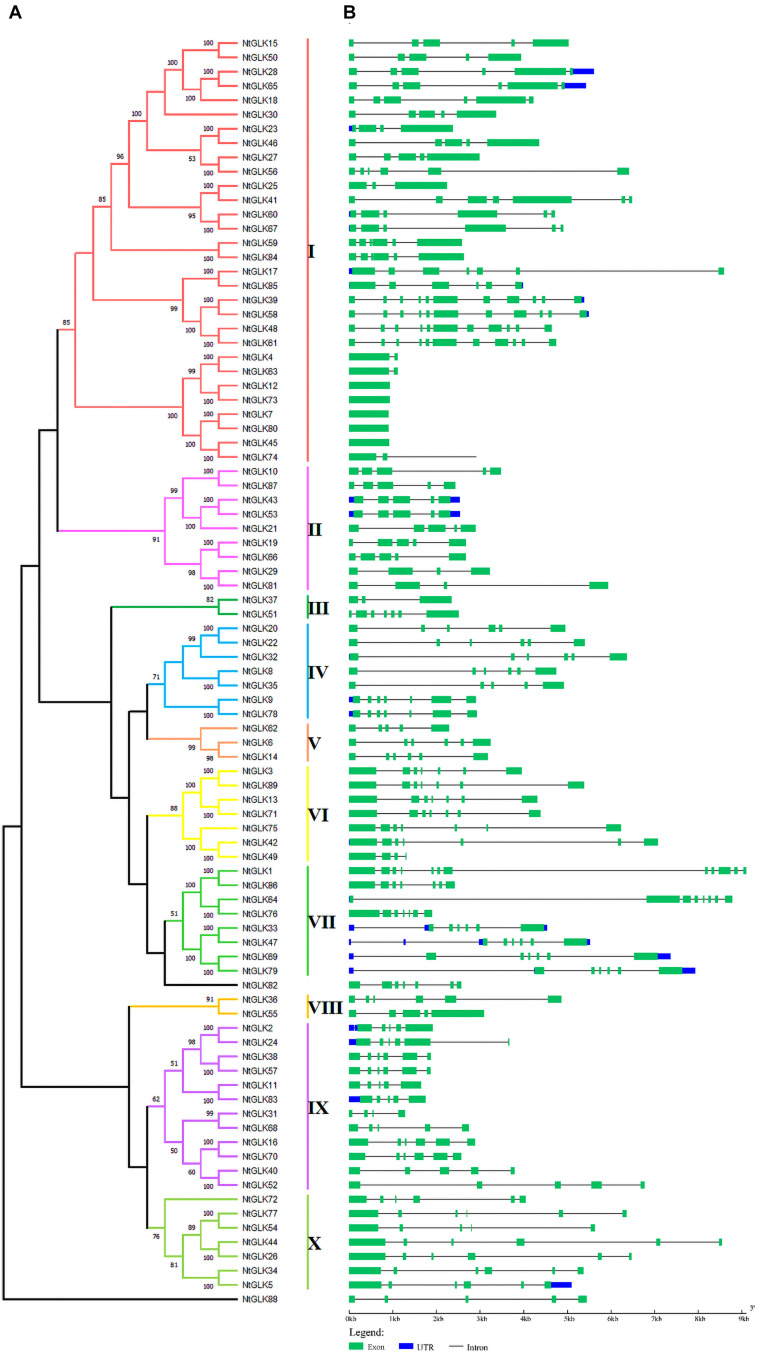
Phylogenetic tree and gene structures of the *NtGLK* gene family. **(A)** A neighbor-joining (NJ) phylogenetic tree was generated by MEGAX based on the NtGLK protein sequences. The different subfamilies are distinguished by *different colors*. **(B)** The exon–intron structures for the *NtGLK* genes were obtained using the online software GSDS. The *horizontal black lines* and the *green boxes* represent introns and exon, respectively, and the lengths of the exons and introns can be estimated using the scale.

To explore the evolutionary relationships among the tobacco *GLK* genes, an unrooted phylogenetic tree was generated using the 89 tobacco GLK protein sequences (Figure 1A); moreover, the gene structures for each *GLK* gene were analyzed (Figure 1B). According to the results of the gene structure and the bootstrap values (>50%) of the phylogenetic tree, the tobacco *GLK* gene family was grouped into 10 subfamilies (I to X). However, there were two genes (*NtGLK82* and *NtGLK88*) that could not be clustered into any of the 10 subfamilies because of their low bootstrap values (<50%). Among these subfamilies, subfamily I (containing 30 members) was the largest group and represented more than 30% of the total *NtGLK* members. In contrast, subfamilies III and VIII only contained two members each.

Structure analysis of the *NtGLK* genes (Figure 1B) showed that the number of introns in the subfamilies ranged from 0 to 11. Among them, five genes (*NtGLK12*, *NtGLK73*, *NtGLK7*, *NtGLK80*, and *NtGLK45*) clustered into subfamily I, which did not contain any intron, and two genes (*NtGLK4* and *NtGLK63*) contained only one intron. Most of the *NtGLK* genes that were clustered into the same phylogenetic groups showed similar exon/intron structures, including the intron numbers and exon length. Variations in the intron number might be one of the key factors that resulted in the diversity of the gene structure and function in the course of evolution.

### Motif Analysis of NtGLK Proteins

**FIGURE 2:**
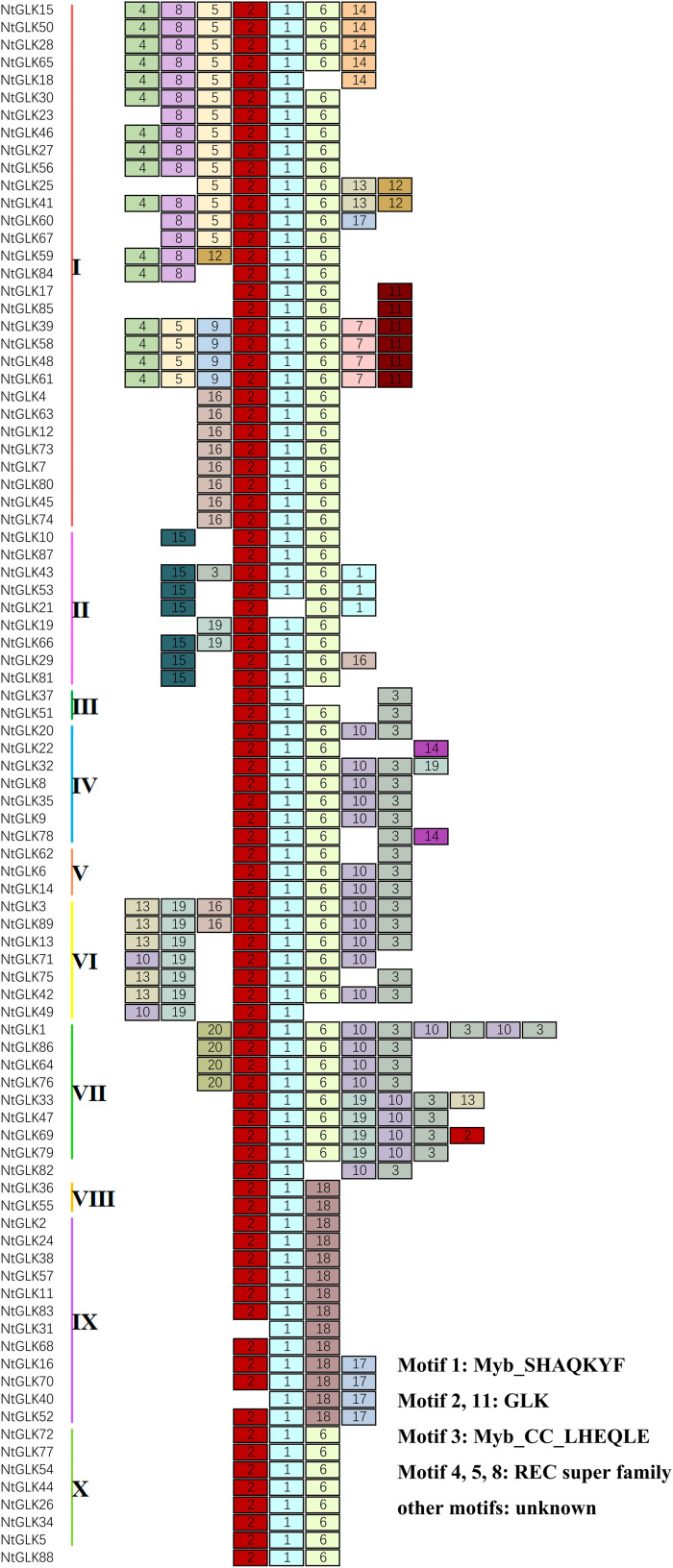
Schematic diagram of the conserved motifs for NtGLK proteins (1–20). The motifs were ordered manually based on the results of the MEME analysis. The annotation information for each motif is shown on the right.

The conserved motifs of the 89 NtGLK proteins within each subfamily were identified and analyzed using the online MEME tool (Figure 2). A total of 20 motifs were identified; the detailed conserved sequences of each motif are shown in Supplementary Table 2. With the CDD tool, seven putative motifs were functionally annotated, which were defined as Myb-SHAQKYF for motif 1, components of the conserved GLK domain for motifs 2 and 11, Myb-CC-LHEQLE for motif 3, and REC superfamily for motifs 4, 5, and 8. However, no functional annotation was assigned for the remaining 13 putative motifs. The motif of Myb-SHAQKYF is highly conserved in a number of myb-related genes. It was reported that the conserved motif of SHAQKYF could bind to the I-box with a DNA-binding domain located in the carboxy terminal domain and acted as a transcriptional activator in yeast and plants (Rose et al., 2000). The motif of Myb-CC-LHEQLE was found toward the C-terminus of Myb-CC-type transcription factors, the member of the protein family reported to be involved in phosphate starvation signaling both in vascular plants and in unicellular algae (Rubio et al., 2001). The functions of the REC domains are annotated as phosphorylation-mediated switches within response regulators (RRs), and some also transfer phosphoryl groups in multistep phosphorelays.

**FIGURE 3:**
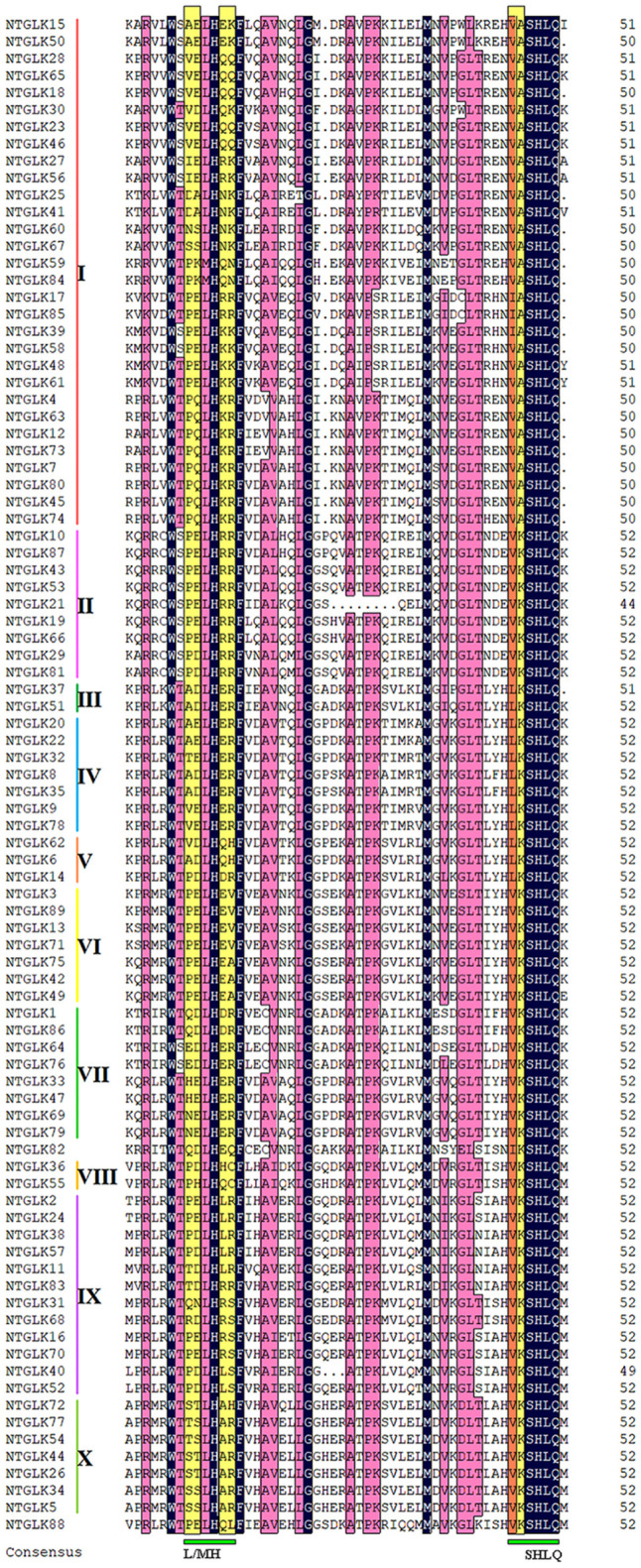
Multiple sequence alignment of the tobacco GLK conserved domain. Identical residues are shaded in black and similar residues in pink.

The majority of the NtGLK proteins contained motifs 1 and 2 (Figure 2). In addition, the NtGLK protein members grouped into the same subfamily contained similar motif components and spatial distributions. Moreover, most of the members of groups III, IV, V, VI, and VII possessed a Myb-CC-LHEQLE domain (motif 3: a type of Myb-like domain), which appeared to respond to various abiotic stresses and played diverse roles in plant development (Murmu et al., 2014). This result suggested that the NtGLK proteins in the same subfamily might have similar functions. In addition, the specificity within a subfamily was also identified. For instance, motif 15 is only possessed by subfamily II and motif 16 only appeared in subfamily I (Figure 2). To further decipher the similarity among the tobacco GLK domains, 89 tobacco GLK domain sequences were aligned using the DNAMAN 8^11^ platform (Figure 3). Our results showed that the GLKs contained two regions of a putative DNA-binding domain with an HLH structure, which was also identified in maize (Liu et al., 2016). In this putative domain, the first helix had initial sequences of PELHRR and the second helix contained the conserved NI/VASHLQ. In addition, although the sequences had particularly conserved L and H, some variants were found for the *GLK* gene members of tobacco in this motif. The second helix region contained a highly conserved sequence of VK/VASHLQ (Liu et al., 2016), which was also similar to the *GLK* members in tomato (Liu, 2018).

### Analysis of *Cis*-Regulatory Elements in the Promoter Regions of *NtGLK* Genes

**FIGURE 4:**
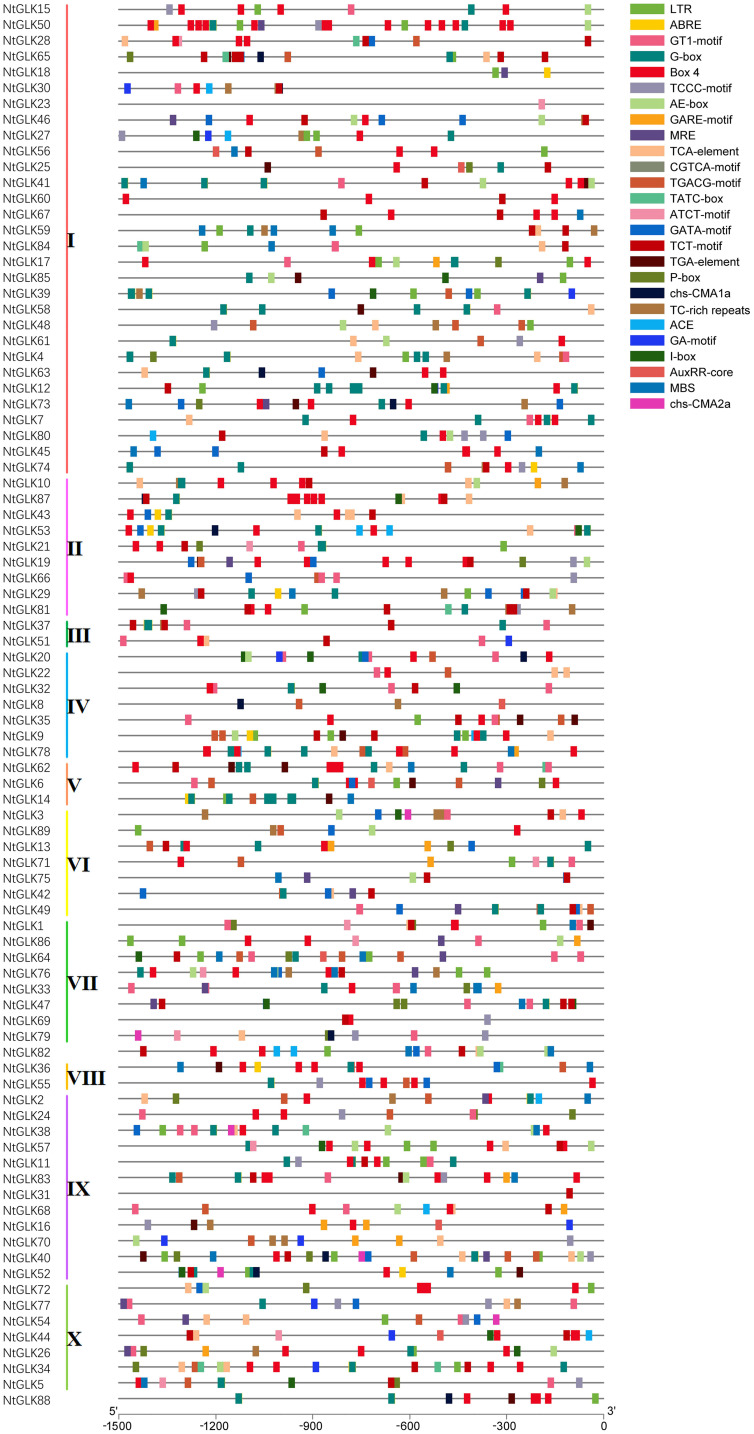
Promoter *cis*-element analysis of *NtGLK* genes. The different types of *cis*-elements are represented by different shapes and colors.

*Cis*-acting elements in the upstream region of the *NtGLK* genes are very important in regulating gene expression in response to various stresses as well as during different developmental stages. To explore the possible expression patterns of the *NtGLK* genes under various stress conditions and senescence processes, *cis*-acting elements including components related to stress, light response, and hormone response were investigated in the promoter regions. Various potential *cis*-acting elements were identified in *NtGLK* promoter regions (Figure 4 and Supplementary Table 3). Among them, the most abundant *cis*-acting elements were light-responsive elements, including GT1-motif, ACE, G-box, ATCT-motif, Box 4, TCT-motif, chs-CMA1a, GATA-motif, I-box, chs-CMA2a, GA-motif, AE-box, MBS, MRE, and TCCC-motif; Box 4 and G-box appear to be the most abundant light-responsive elements, being distributed in the promoter regions of 65 and 56 *NtGLK* genes, respectively. In terms of the hormone response-related *cis*-acting elements, a total of nine types of elements were identified, namely, TGA-element, AuxRR-core, TCA-element, ABRE, CGTCA-motif, TGACG-motif, P-box, GARE-motif, and TATC-box; among them, ABRE was the most abundant *cis*-acting hormone-responsive element in the promoter regions of 89 *NtGLK* genes, which was involved in abscisic acid (ABA) responsiveness. This abundance of hormone-responsive elements indicated that *NtGLK* genes appeared to play important roles in tobacco hormone signal transduction and senescence. In addition, a total of two kinds of *cis*-acting elements involved in various stresses were found, including long terminal repeat (LTR) in low-temperature responsiveness and TC-rich repeats in defense and stress responsiveness (Supplementary Table 3). Notably, many *cis*-elements have two or more copies in the 1.5-kb upstream region within the same *NtGLK* gene, which appear to enhance their binding effects to their corresponding *trans*-acting factors. Furthermore, the *NtGLK* genes in the same phylogenetic clade only showed moderate consistency in their distributions of the *cis*-elements, reflecting the complex evolutionary relationship of the diverged *NtGLK* genes, especially in promoter regions.

### Chromosomal Locations and Duplications of *NtGLK* Genes

**FIGURE 5:**
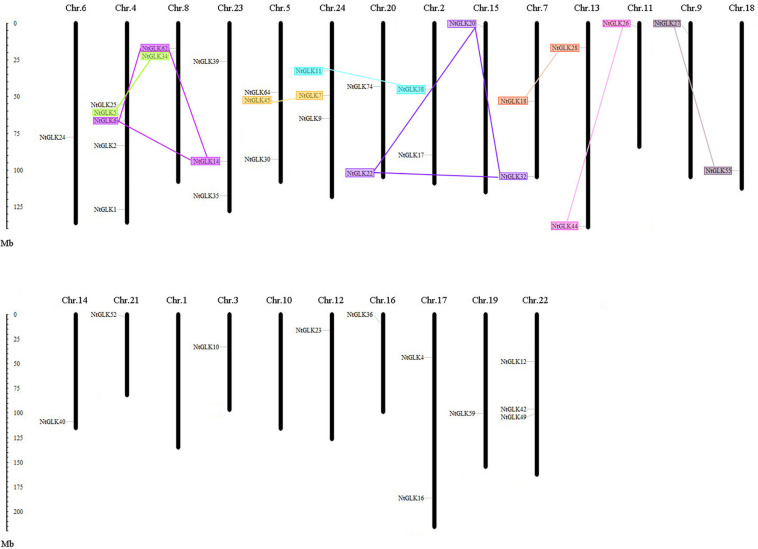
Chromosomal locations of tobacco *GLK* genes. Segmented duplicated gene pairs are displayed as color boxes connected by lines.

Except for chromosomes 1 and 10, 40 out of the 89 *NtGLK* genes were obtained and were assigned to the 22 tobacco chromosomes (Figure 5). Chromosome 4 contained the largest number of *NtGLK* genes (five), and chromosomes 3, 6, 9, 11, 12, 14, 15, 16, 18, 19, and 21 possessed only one *NtGLK* gene each. A similar uneven distribution pattern of the *GLK* gene family was also found in maize (Liu et al., 2016). In addition, the potential duplication events were investigated to explore the potential mechanism for the *NtGLK* gene family. A total of 12 duplicated pairs of *NtGLK* genes were identified as segmental duplication gene pairs (Figure 5 and Supplementary Table 4). However, no pairs of tandem duplicated genes were observed in this study. A biased distribution pattern was also found among the 12 segmental duplication pairs, and no pairs were distributed on chromosomes 1, 3, 6, 10, 12, 14, 16, 17, 19, 21, and 22. These results infer that segmental duplication events may play important roles in the amplification of the tobacco *GLK* gene family.

### Phylogenetic Analysis of the *NtGLK* Gene Family

**FIGURE 6:**
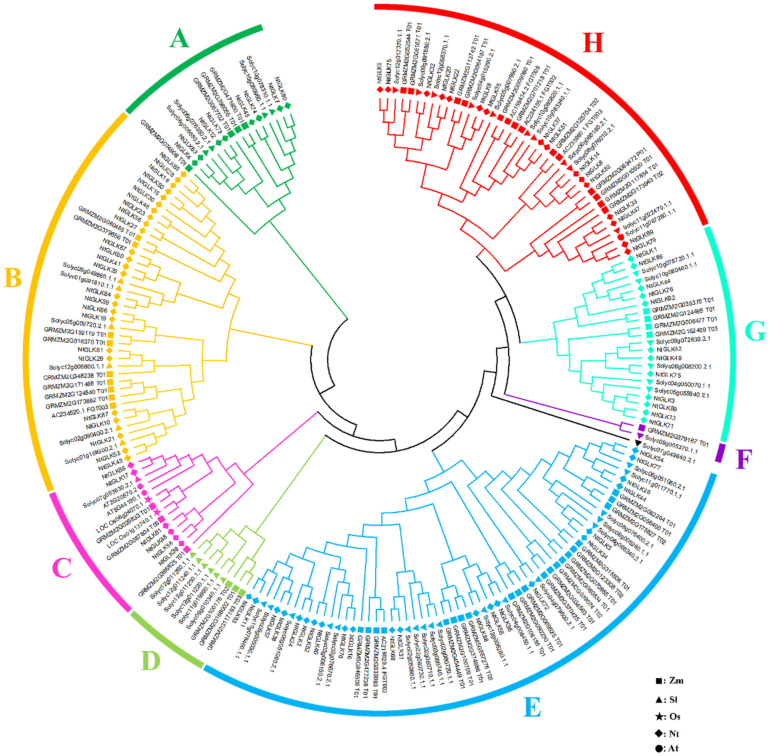
Phylogenetic analysis of tobacco, maize, rice, *Arabidopsis*, and tomato GLK proteins. Different GLK subfamilies are shown as different colors. The square, triangle, star, diamond, and circle represent maize, tomato, rice, tobacco, and *Arabidopsis* GLK proteins, respectively.

To analyze the evolutionary process of the tobacco *GLK* gene family, the 89 NtGLK protein sequences were aligned with 59, 54, two, and two GLK proteins from maize (Liu et al., 2016), tomato (Liu et al., 2016), rice (Rossini et al., 2001), and *Arabidopsis* (Fitter et al., 2002), respectively. In total, 206 GLK proteins were clustered into eight groups (A to H; Figure 6). Group E was the largest subfamily, which contained 61 proteins, including 22 from tobacco, 18 from tomato, and 21 from maize. Groups B (40) and H (41) also had large numbers of GLK members, and these three groups represented 68.9% of the total NtGLK proteins. In contrast, group F was the smallest clade, which had only two *GLK* gene members. Notably, group C contained 14 GLK members, and among them, two genes (*At5G44190.1* and *At2G20570.2*) were confirmed to be involved in leaf senescence in *Arabidopsis* (Rauf et al., 2013). It appears that the members clustered into group C, including six members from tobacco (*NtGLK85*, *NtGLK17*, *NtGLK61*, *NtGLK48*, *NtGLK58*, and *NtGLK39*) may share similar functions. In addition, the GLK members from tobacco, tomato, and maize were distributed into the major subfamilies, suggesting that the *GLK* gene family existed before the separation of monocotyledons and dicotyledons.

### Expression Patterns of *NtGLK* Genes in Different Senescence Stages of Tobacco Leaves

**FIGURE 7:**
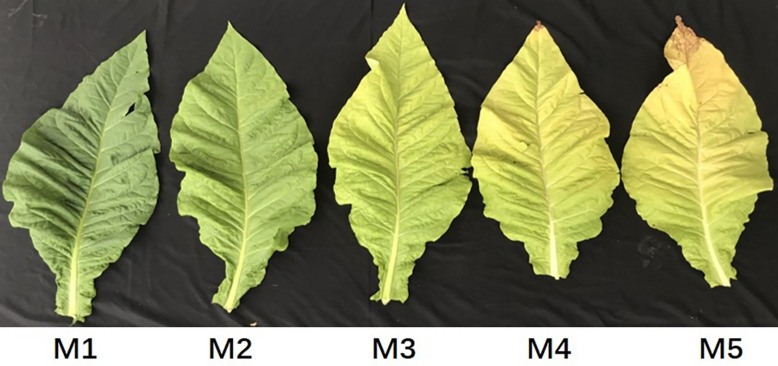
Tobacco leaves with different senescence degrees.

**FIGURE 8:**
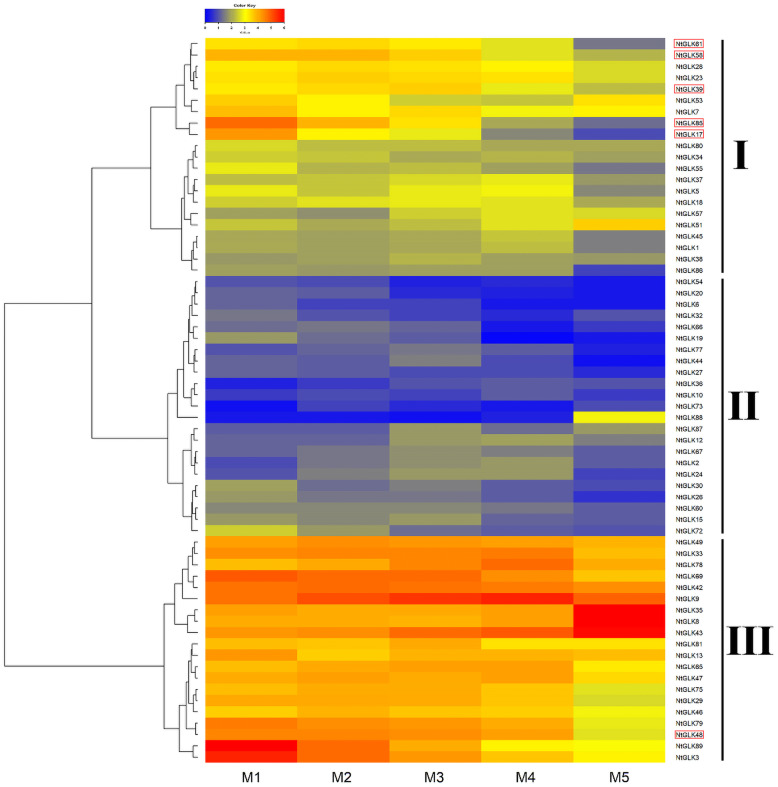
Expressions of *NtGLK* genes in tobacco leaves at different senescence stages.

Tobacco leaves at the M1, M2, M3, M4, and M5 senescence stages were collected, which had yellowing rates of 50, 65, 75, 85, and 100%, respectively (Figure 7). To explore the potential functions of the *NtGLK* genes, an RNA sequencing (RNA-Seq) experiment was performed for tobacco leaves in the five senescence stages (M1–M5), and the FPKM values of the *NtGLK* genes derived from the RNA-Seq data were used to evaluate the expression levels of these *NtGLK* genes in the different senescence stages. Among them, 25 *NtGLK* genes were excluded from further heat map analysis because of low expression (FPKM < 0.5) or lack of expression in all the senescence stages. The *NtGLK* genes showed differential expressions in tobacco leaves at the different senescence stages (Figure 8). A total of 64 *NtGLK* genes were clustered into three groups (Figure 8). Among them, 20 genes (*NtGLK49*, *NtGLK33*, *NtGLK78*, *NtGLK69*, *NtGLK42*, *NtGLK9*, *NtGLK35*, *NtGLK8*, *NtGLK43*, *NtGLK81*, *NtGLK13*, *NtGLK65*, *NtGLK47*, *NtGLK75*, *NtGLK29*, *NtGLK46*, *NtGLK79*, *NtGLK48*, *NtGLK89*,and *NtGLK3*) were included in group III (Figure 8), which exhibited high expression levels in all the analyzed stages, hinting that these genes were essential in the five senescence stages of tobacco leaves. However, 23 *NtGLK* genes (*NtGLK54*, *NtGLK20*, *NtGLK6*, *NtGLK32*, *NtGLK66*, *NtGLK19*, *NtGLK77*, *NtGLK44*, *NtGLK27*, *NtGLK36*, *NtGLK10*, *NtGLK73*, *NtGLK88*, *NtGLK87*, *NtGLK12*, *NtGLK67*, *NtGLK2*, *NtGLK24*, *NtGLK30*, *NtGLK26*, *NtGLK60*, *NtGLK15*, and *NtGLK72*) were clustered into group II and showed relatively lower expression levels in all stages. In general, the expression levels of most *NtGLK* genes exhibited a decreased trend with the increase of senescence level, such as *NtGLK89*, *NtGLK3*, *NtGLK85*, *NtGLK17*, *NtGLK58*, etc. However, inverse expression patterns were also found in some *NtGLK* genes, such as *NtGLK9*, *NtGLK35*, *NtGLK8*, and *NtGLK43*. It is worth noting that *NtGLK85*, *NtGLK17*, and *NtGLK58* were all grouped into subfamily I, and *NtGLK9*, *NtGLK35*, and *NtGLK8* were all clustered into subfamily IV (Figure 1). The results showed that the expression patterns of the *NtGLK* genes in the different senescence stages were different. The expression patterns of the *NtGLK* genes provided preliminary information for their further functional exploration.

**FIGURE 9:**
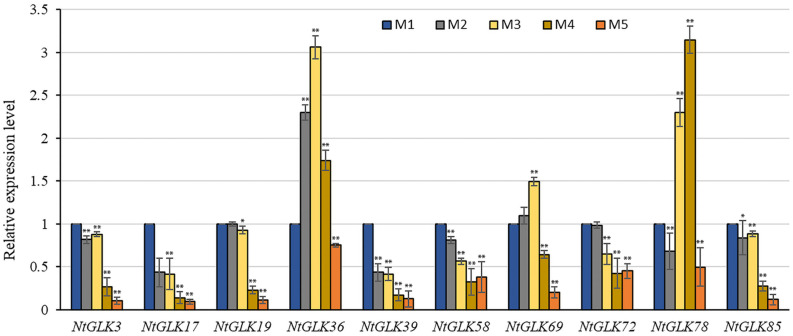
Relative expression levels of 10 *NtGLK* genes in tobacco leaves at five senescence stages. Error bars indicate standard deviation. Asterisks indicate the significant degree of expression level compared to the value of the control (^∗^*P* < 0.05, ^∗∗^*P* < 0.01).

To validate the RNA-Seq data, 10 *NtGLK* genes were selected for qRT-PCR analysis (Supplementary Table 1). Among them, variations of the transcript abundances of six genes (*NtGLK*3, *NtGLK19*, *NtGLK36*, *NtGLK69*, *NtGLK72*, and *NtGLK78*) corresponded to the increase of the senescence degrees based on the RNA-Seq data. Another four *GLK* genes (*NtGLK85*, *NtGLK17*, *NtGLK58*, and *NtGLK39*) and two Arabidopsis genes (*At5G44190.1* and *At2G20570.2*) were grouped together (group C; Figure 6), and these two *Arabidopsis* genes were previously confirmed to be involved in leaf senescence (Rauf et al., 2013). The results of qRT-PCR showed that the expression levels of *NtGLK3*, *NtGLK17*, *NtGLK19*, *NtGLK39*, *NtGLK58*, *NtGLK72*, and *NtGLK85* were decreased, precisely consistent with the increase of the senescence degrees, while the expressions of *NtGLK36*, *NtGLK69*, and *NtGLK78* showed a trend of rising first and then decreasing (Figure 9). The expression patterns of these 10 genes detected by qRT-PCR showed similar trends of gene expression pattern to those detected by the RNA-Seq approach (Figure 8), indicating that the RNA-Seq data were reliable.

## Discussion

The function of *GLK* genes was first recognized from the analysis of a maize mutant with pale green phenotype (Langdale and Kidner, 1994) and acted as a transcription regulatory element (Hall et al., 1998). The *GLK* gene sequences have only been identified in photosynthetic eukaryotes such as green algae and higher plants, but absent in the genome of the cyanobacterium *Synechocystes*, suggesting that the function of *GLK* genes is associated with the development of chloroplasts (Rossini et al., 2001). Members of the *GLK* gene family are considered as one of the most characterizable genes among the nuclear-encoded genes, which regulate chloroplast biogenesis (Fitter et al., 2002). Chloroplast converts light energy into chemical energy, and it plays important roles in the growth and development of plants (Kirchhoff, 2019). The color of leaves gradually changes during leaf senescence with the chlorophyll broken down and the photosynthetic capacity declined (Lira et al., 2017; Schippers et al., 2015; Gan and Amasino, 1997; Lim et al., 2003); therefore, the leaf senescence of tobacco can be assessed by the degree of leaf etiolation. Two *Arabidopsis* genes (*At5G44190.1* and *At2G20570.2*) were confirmed to be involved in leaf senescence (Rauf et al., 2013). Generally, genes that belong to the same subfamily share similar functions compared to those from different subfamilies. In our analysis, a total of six tobacco *GLK* genes (*NtGLK85*, *NtGLK17*, *NtGLK39*, *NtGLK48*, *NtGLK58*, and *NtGLK61*) were clustered with the *Arabidopsis GLK* genes (*At5G44190.1* and *At2G20570.2*) in group C (Figure 6), suggesting that these genes across different species might share structural and functional similarity. Thus, it has been postulated that these six putative *NtGLK* genes play roles in the leaf senescence of tobacco. According to the FPKM values generated from the RNA-Seq data, we found that the transcript abundance of the six *NtGLK* genes in subfamily C decreased, closely corresponding to the increase of leaf senescence level, indicating that these six *NtGLK* genes might play important roles in the process of tobacco leaf senescence. The harvest maturity of tobacco leaf is closely related to leaf senescence, and it is considered as a fundamental index to measure tobacco quality. To improve the quality of tobacco, it is very important to develop proper parameters for harvest maturity. Hence, the variations of the transcript abundance of these six *GLK* genes can be further developed as markers to evaluate the maturity of tobacco leaves.

The *NtGLK* genes are not evenly distributed in the tobacco genome. A similar uneven distribution pattern of the GLK gene family was also identified in maize (Liu et al., 2016). Unlike prokaryotes, it is common that functionally related genes in eukaryotes are distributed throughout genomes. The physical arrangement of genes on chromosomes is largely derived from insertions, deletions, duplications, and inversions. As a regulatory mechanism of gene expression in cells, microRNAs (miRNAs) target specific messenger RNAs (mRNAs) to regulate gene expression through the mechanism of RNA interference (RNAi). It was observed that the miRNA counts were extremely high in certain chromosomes for the 20 different species, suggesting that the higher number of miRNA genes in these chromosomes might be associated with a regulatory role in cellular functions (Atanu and Utpal, 2014). The co-localized and “operon-like” biosynthetic gene clusters have also been identified in eukaryotes with unknown mechanisms related to highly interactive domains for the regulation of co-expression within clusters of genes (Nützmann et al., 2020). Therefore, the cluster of *NtGLK* may be related to the regulation of genes with leaf senescence.

As the transcription factor, the *G2* gene is activated by the N-terminal region of a heterologous system, and the *GLK* gene family members contain the highly conserved HLH region (DNA-binding domain) and the GCT box (which functions in dimerization) (Rossini et al., 2001). The sequence of the DNA-binding domain belongs to the GARP transcription factor family (Hosoda et al., 2002), and the two regions of the HLH DNA-binding domain are also conserved (Liu et al., 2016). These two conserved regions of HLH structure were also identified in the tobacco genome in this study; however, the conserved sequences were not totally identical in tobacco. Multiple sequence alignment demonstrated that the second helix region of the *NtGLK* genes was highly conserved (VK/VASHLQ), while a number of variants were found for the *NtGLK* genes in the first helix, except for the fairly conserved L and H, suggesting that the first helix appeared to be more important in the functional differentiation of the *GLK* gene family in tobacco. This diversity in function derived from sequence variants was also observed in maize research, where the *ZmGLK* genes were overall much conserved within the course of evolution (Liu et al., 2016). Actually, the genetic variants derived from the single nucleotide polymorphisms (SNPs) were responses for the gene function of cultivar adatation in tropical and temperate lines, which are linked to resistance to cold and drought stresses (Liu et al., 2016). In our study, the number of variants in each subfamily seemed different among the 10 subfamilies of the *GLK* gene family in tobacco (Supplementary Table 5), and subfamilies I, IV, VII, and IX showed more sequence variants. The results suggest that sequence diversity was the important factor leading to the more diverse functions in subfamilies I, IV, VII, and IX than in the other groups.

Multigenic families are usually derived from gene duplications, and the expansion mechanism includes unequal crossing-over, various transposition events, duplication of large chromosome segments, or polyploidization events (Zhang, 2003; Passardi et al., 2004). Theoretically, duplication events can often produce two gene copies, and one or both copies can acquire novel functions under a smaller selective pressure of evolution (Van de Peer et al., 2009). It appears that the loss and the insertion of new introns are frequent events, and they play important roles in gene evolution. It was reported that the number of introns was largely reduced and less frequently gained in eukaryotes in the course of evolution (Rogozin et al., 2012). In addition, analysis of segmental duplication events in rice showed that more introns were lost than gained (Lin et al., 2006). In this study, the intron distribution within *NtGLK* genes is quite variable, and the range varied from 0 to 11 (Figure 1), inferring that the shuffling of introns has been a main configuration for the evolution of *NtGLK* genes since their origin. Moreover, a total of four duplication gene pairs, including *NtGLK6/62*, *NtGLK14/62*, *NtGLK11/38*, and *NtGLK27/55* (Supplementary Table 4), appeared to have experienced intron loss events based on our analysis. Notably, one of the transposition events, the retrotransposition of cDNA, is characterized by the loss of all introns and regulatory sequences and by a random insertion within the genome (Casacuberta and Santiago, 2003). In this study, there were five *NtGLK* genes with no introns (*NtGLK12*, *NtGLK73*, *NtGLK7*, *NtGLK80*, and *NtGLK45*), suggesting that these genes might be derived from the retrotransposition events. One of the gene pairs with no introns (*NtGLK7*/*NtGLK45*) met the parameters of segmental duplication; thus, they might be due to retrotransposition, but not originated from segmental duplication.

The *GLK* genes have been derived from independent gene duplication as a group of pairs in plants including monocots, eudicots, and bryophytes, but the regulation of *GLK* gene expression appears to be different (Yasumura et al., 2005). The *GLK* genes in maize act differentially in mesophyll cells and bundle sheath for chloroplast development, while they direct the development of chloroplasts in *Arabidopsis* to be monomorphic (Rossini et al., 2001; Fitter et al., 2002). To explore the potential functions of the *NtGLK* genes, RNA-Seq (Figure 8) and qRT-PCR for tobacco leaves in five senescence stages (Figure 9) were conducted for gene expression analyses. The expression levels of some *NtGLK* genes were decreased or increased, precisely corresponding consistently to the increase of the senescence degrees. Whether or not the differential expressions of *GLK* genes lead to assessing thylakoid deformation and impaired chlorophyll biosynthesis in chloroplasts still needs to be investigated. Studies with mutant and cross-species complementation experiments have demonstrated that, although *GLK* gene functions appear to be conserved in different species, the cross-species regulatory elements cannot drive gene expression in other species, suggesting that the *GLK* functional pathway has been diverged and species-specific during land colonization (Bravo-Garcia et al., 2009). Therefore, analysis of the specific expression patterns for these *NtGLK* genes provide preliminary information for their further functional exploration.

## Conclusion

In this study, a total of 89 *NtGLK* genes were identified in the tobacco genome. They were classified into 10 subfamilies with diverse structures. Twelve pairs of *NtGLK* genes were found to be originated from segmental duplication. Phylogenetic analysis of the *NtGLK* genes showed that the *GLK* gene family existed prior to the separation of monocotyledons and dicotyledons. The *NtGLK* genes showed differential expression patterns in tobacco leaves at five senescence stages; among them, the expression levels of six genes (*NtGLK85*, *NtGLK17*, *NtGLK39*, *NtGLK48*, *NtGLK58*, and *NtGLK61*) were reduced, coinciding precisely with the increment of the degree of senescence, suggesting that these genes can be further developed as marker genes for maturity evaluation. Our results provide valuable information for further functional study of the *NtGLK* genes.

## Data Availability Statement

The raw data supporting the conclusions of this article will be made available by the authors, without undue reservation.

## Author Contributions

XX designed this research and wrote the manuscript. MQ and BZ performed the experiments and analyzed the data. GG and XY collected the plant materials and performed the experiments. JZY helped draft the manuscript. JHY participated in handling the figures and tables. All authors contributed to the article and approved the submitted version.

## Conflict of Interest

The authors declare that the research was conducted in the absence of any commercial or financial relationships that could be construed as a potential conflict of interest.

## Footnotes

1 https://solgenomics.net/organism/Nicotiana_attenuata/genome

2 https://www.ncbi.nlm.nih.gov/Structure/cdd/wrpsb.cgi

3 https://solgenomics.net

4 http://web.expasy.org/protparam/

5 http://dnaman.software.informer.com/

6 http://www.plantbreeding.wur.nl/uk/software_mapinspect.html

7 http://gsds.cbi.pku.edu.cn/

8 http://alternate.meme-suite.org/tools/meme

9 http://bioinformatics.psb.ugent.be/webtools/plantcare/html/

10 http://www.biomarker.com.cn/

11 http://dnaman.software.informer.com/
