# Supplementary material for: Genome-Wide Analysis of the G2-Like Transcription Factor Genes and Their Expression in Different Senescence Stages of Tobacco (Nicotiana tabacum L.)
Source: Front Genet. 2021 May 31;12:626352. doi: 10.3389/fgene.2021.626352 (PMC8202009; doi:10.3389/fgene.2021.626352)
Supplement: Supplementary file 1 [file Table_1.DOCX]

| **Table S1.** Tobacco *GLK* gene-specific primers used for qRT-PCR analysis. | | |
| --- | --- | --- |
| Primer Name | Forward Primer (5′→3′) Sequence | Reverse Primer (5′→3′) Sequence |
| NtGLK3 | CGAAAAACATGGGGAAAGAA | GGCGACTTTCGTTCATTGTT |
| NtGLK17  NtGLK19 | TGCTTGCTGTGTCATCTTTGA  CTCATGTTGCTACGCCAAAA | TGGTAGCAAATCTCCGTCGT  TGTGGTGGTTGTTGGTTGTT |
| NtGLK36 | TCGAGGGCTCAAGGAAAATA | TCGTGATGATTCAGGTTGGA |
| NtGLK39 | GTGCCAAAGATCTGCCAACA | TTGGTTTAAGCGATTCGGGC |
| NtGLK58 | CTTGGGTACCACCAGCAAGT | TCCTAATGATGGTGGCATGA |
| NtGLK69 | AGCAACTGGAGGTCCAGAAA | AGATTCTGGGCCATTGTCAC |
| NtGLK72 | TAGGTGGACATGAACGAGCA | TTCCCTCTGTTTGGGTTCAC |
| NtGLK78 | CTTCACGAGCGTTTTGTTGA | TGAGGTTGCTTTCCCAGTCT |
| NtGLK85 | TTCCCAAACCTCCTTGTGAC | ACTGCGCTGCCTCAATAACT |
| NtActin | ACCTCTATGGCAACATTGTGCTCAG | CTGGGAGCCAAAGCGGTGATT |
