# Supplementary material for: Genome-Wide Analysis of the G2-Like Transcription Factor Genes and Their Expression in Different Senescence Stages of Tobacco (Nicotiana tabacum L.)
Source: Front Genet. 2021 May 31;12:626352. doi: 10.3389/fgene.2021.626352 (PMC8202009; doi:10.3389/fgene.2021.626352)
Supplement: Supplementary file 2 [file Table_2.DOCX]

**Table S2.** Sequences of 20 predicted motifs of StPHD proteins

| **Motif** | | **Width** | **Motif Sequence** | **Annotation** |
| --- | --- | --- | --- | --- |
| 1 | 31 | | LGGIDKATPKTILZLMNVKGLTREHVKSHLQ | Myb_SHAQKYF |
| 2 | 22 | | TSKKPRLRWTPELHERFVDAVE | GLK |
| 3 | 31 | | QKRLHEQLEIQRKLQLRIEAQGKYLQMIFEK | Myb_CC_LHEQLE |
| 4 | 59 | | VLVVDDDPTCLKILEKMLRKCHYEVTTCNRAEEALSLJRENKNGFDLVISDVHMPBMDG | REC super family |
| 5 | 31 | | HGACDYLIKPIRDEELKNIWQHVVRKKKNEG | REC super family |
| 6 | 12 | | KYRLYKKRPSPS | – |
| 7 | 84 | | YPPHKPIMAFPPYHSNHAPAAGQFYPAWVPPGSYPNGLQVWGSPYYPGWQPADSWHWKPHPGLHADVWGSPVTPPSFGSYPPYP | – |
| 8 | 22 | | FKLLEHIGLEMDLPVIMMSADD | REC super family |
| 9 | 100 | | CDKYPAPSTPQQKQGVRSVDDGDFQDHTIFSTEQDSGENDGDTKSVETTFNDSVAETTVQTDPPGRQGZAITKEENGSAPDQKMEDDIATSSPSNDCPGN | – |
| 10 | 16 | | KTGMZITEALKMQMEV | – |
| 11 | 60 | | NRYSLLEKSFDLHPAEEVIDKVVKEAISKPWLPLPLGLKPPSTDSVLGELSRQGISTVPP | GLK |
| 12 | 31 | | YLNQGANRMQLTRLPNNVETGGGSGTKPGQD | – |
| 13 | 60 | | SNSGTVGHLLSSSSGPHKDLHFSPTSPQESRPRPYPFISSSASAETSQASPSSLVSTSLA | – |
| 14 | 62 | | NSGMSTLTSKGMLQEEVNSDIRGSRGFPSNYDMFDELHQQKSQDWGLQNVGSTFDASHHSSI | – |
| 15 | 37 | | EYVARLEEERKKIDAFKRELPLCMLLVTDAIEALRQZ | – |
| 16 | 31 | | LPSPEELTPLSQTLIPPELASAFDISPETSP | – |
| 17 | 43 | | HRAMQGKSYFYGNVGQRYNPLQDFKMKNGAIVLARNFNYDDDH | – |
| 18 | 12 | | MYRSKKJDDPGQ | – |
| 19 | 17 | | KRSDWQEWADQLINDDD | – |
| 20 | 60 | | MSIQRFGTQDRVPKTNEYASDYTLEFPKIPVQDLVAQKQYSSHMGFSFPPEKSRZZQHQQ | – |

–Means no annotation was found
