# Supplementary material for: Genome-Wide Analysis of the G2-Like Transcription Factor Genes and Their Expression in Different Senescence Stages of Tobacco (Nicotiana tabacum L.)
Source: Front Genet. 2021 May 31;12:626352. doi: 10.3389/fgene.2021.626352 (PMC8202009; doi:10.3389/fgene.2021.626352)
Supplement: Supplementary file 5 [file Table_5.DOCX]

**Table S5.** Variants of the two conserve regions in NtGLK proteins

| Subfamily | Type | Region 1(PELHRR) | Region 2 (VASHLQ) |
| --- | --- | --- | --- |
| Ⅰ | 1 | AELHEK | VASHLQ |
|  | 2 | VELHQQ |  |
|  | 3 | VDLHQK |  |
|  | 4 | IELHRK |  |
|  | 5 | DALHNK |  |
|  | 6 | NSLHNK |  |
|  | 7 | SSLHNK |  |
|  | 8 | PKMHQN |  |
|  | 9 | PQLHKR |  |
| Ⅱ | 1 | PELHRR | VKSHLQ |
|  | 2 | PDLHRR |  |
| Ⅲ | 1 | PERHLF | LKSHLQ |
| Ⅳ | 1 | AELHER | LKSHLQ |
|  | 2 | TELHER |  |
|  | 3 | ADLHER |  |
|  | 4 | VELHER |  |
| Ⅴ | 1 | PRLRWT | LKSHLQ |
| Ⅵ | 1 | PELHEV | VKSHLQ |
|  | 2 | PELHEA |  |
| Ⅶ | 1 | QDLHDR | VKSHLQ |
|  | 2 | EDLHER |  |
|  | 3 | HELHER |  |
|  | 4 | NELHER |  |
| Ⅷ | 1 | PDLHHC | VKSHLQ |
|  | 2 | PHLHQC |  |
| Ⅸ | 1 | PELHLR | VKSHLQ |
|  | 2 | PDLHLR |  |
|  | 3 | TDLHRR |  |
|  | 4 | TDLHRR |  |
|  | 5 | QNLHRS |  |
|  | 6 | RDLHRS |  |
|  | 7 | PELHRS |  |
|  | 8 | PDLHLS |  |
| Ⅹ | 1 | STLHAH | VKSHLQ |
|  | 2 | TSLHAR |  |
|  | 3 | STLHAR |  |
|  | 4 | SSLHARR |  |
